# Effect of CuO and Graphene on PTFE Microfibers: Experimental and Modeling Approaches

**DOI:** 10.3390/polym14061069

**Published:** 2022-03-08

**Authors:** Maroof A. Hegazy, Hend A. Ezzat, Ibrahim S. Yahia, Heba Y. Zahran, Hanan Elhaes, Islam Gomaa, Medhat A. Ibrahim

**Affiliations:** 1 Nano Unit, Space Lab, Solar and Space Research Department, National Research Institute of Astronomy and Geophysics (NRIAG), Helwan 11421, Egypt; maroof.hegazy@gmail.com (M.A.H.); hend.ezzat@nriag.sci.eg (H.A.E.); 2 Laboratory of Nano-Smart Materials for Science and Technology (LNSMST), Department of Physics, Faculty of Science, King Khalid University, P.O. Box 9004, Abha 61413, Saudi Arabia; dr_isyahia@yahoo.com (I.S.Y.); dr_hyzahran@yahoo.com (H.Y.Z.); 3 Research Center for Advanced Materials Science (RCAMS), King Khalid University, P.O. Box 9004, Abha 61413, Saudi Arabia; 4 Nanoscience Laboratory for Environmental and Bio-Medical Applications (NLEBA), Semiconductor Lab., Metallurgical Lab. 1., Physics Department, Faculty of Education, Ain Shams University, Roxy 11757, Egypt; 5 Physics Department, Faculty of Women for Arts, Science and Education, Ain Shams University, Cairo 11757, Egypt; hanan.elhaes@women.asu.edu.eg; 6 Nanotechnology Research Centre (NTRC), The British University in Egypt (BUE), Suez Desert Road, El-Sherouk City 11837, Egypt; islam.gomaa@bue.edu.eg; 7 Molecular Spectroscopy and Modeling Unit, Spectroscopy Department, National Research Centre, 33 El-Bohouth St., Dokki 12622, Egypt

**Keywords:** PTFE microfibers, FTIR, XRD, FESEM, G, DFT, metal oxides

## Abstract

The surface of pure polytetrafluoroethylene (PTFE) microfibers was modified with ZnO and graphene (G), and the composite was studied using ATR-FTIR, XRD, and FESEM. FTIR results showed that two significant bands appeared at 1556 cm^−1^ and 515 cm^−1^ as indications for CuO and G interaction. The SEM results indicated that CuO and G were distributed uniformly on the surface of the PTFE microfibers, confirming the production of the PTFE/CuO/G composite. Density functional theory (DFT) calculations were performed on PTFE polymer nanocomposites containing various metal oxides (MOs) such as MgO, Al_2_O_3_, SiO_2_, TiO_2_, Fe_3_O_4_, NiO, CuO, ZnO, and ZrO_2_ at the B3LYP level using the LAN2DZ basis set. Total dipole moment (TDM) and HOMO/LUMO bandgap energy ΔE both show that the physical and electrical characteristics of PTFE with OCu change to 76.136 Debye and 0.400 eV, respectively. PTFE/OCu was investigated to observe its interaction with graphene quantum dots (GQDs). The results show that PTFE/OCu/GQD ZTRI surface conductivity improved significantly. As a result, the TDM of PTFE/OCu/GQD ZTRI and the HOMO/LUMO bandgap energy ΔE were 39.124 Debye and ΔE 0.206 eV, respectively. The new electrical characteristics of PTFE/OCu/GQD ZTRI indicate that this surface is appropriate for electronic applications.

## 1. Introduction

PTFE, one of several synthetic polymeric matrices, has excellent corrosion resistance and electrical properties, as well as high temperature resistance and cost-effectiveness [1,2]. Polymers of various forms, particularly fibers influenced by MOs, are frequently used to improve polymer characteristics, resulting in low-cost, high-functioning nano-composites [3,4,5,6]. Because of the mechanical, physical, and chemical stability of PTFE, it may be utilized as a substrate for the development of ZnO nanotubes, allowing for the effective production of various sensors [7] and nanoscale photodetectors used for nano optics applications [8]. Furthermore, because of the unique properties of SiO_2_ nanoparticles, such as high hardness, corrosion resistance, and superior electrical insulation, PTFE/SiO_2_ might be used in technological applications [9] and for vapor oil purification [10]. Moreover, by increasing the SiO_2_ amount, the PTFE/SiO_2_ composite possesses good mechanical characteristics [11]. PTFE/SiO_2_/Epoxy composites provide a new hybrid composite with unique properties [12]. The PTFE/Al_2_O_3_ nanocomposite shows a high mechanical characteristic with increased thermal conductivity and thermal stability [13,14]. Furthermore, the electrical characteristics of the PANI/PTFE/GO composite have been observed to have increased in the fabrication of electrochemical instruments [15]. Further, the corrosion resistance and insulating characteristics of PTFE/ZnO/SiO_2_ on glass have already been considerably enhanced, and this technique provides a novel idea for building an insulator surface on glass that acts as an anti-icing surface [16]. Additionally, there is a Teflon FEP derivative of PTFE that is employed as a thermal control barrier for the Hubble Space Telescope (HST) [17,18,19]. In a space environment in Low Earth orbit (LEO), Teflon FEP suffers from corrosion, exposing the component in space to damage [20,21,22]. As a result, the improvement of Teflon and its derivatives has become an important research topic for space applications [23,24,25]. Physical parameters determined using simulations, such as TDM, HOMO/LUMO band gap energy (ΔE), and MESP, are thought to be effective indicators of electronic properties and the responsiveness of such explored interactions [26,27,28]. In addition, it was shown that reactive systems have a high TDM when the energy band gap is minimal, and the charge distribution as contour of the MESP can be compared with the corresponding active sites along the base material [29,30,31,32]. 

In this work, we investigate the effect of ZnO and G on PTFE microfibers, which are characterized to investigate changes in molecular structure, crystal structure, and morphology. Moreover, in order to study the influence of MOs such as MgO, Al_2_O_3_, SiO_2_, TiO_2_, Fe_3_O_4_, NiO, CuO, ZnO, and ZrO_2_ on PTFE electronic characteristics, it is required to analyze the change in TDM, band gap, and MESP. The goal of this theoretical work is to elucidate the influence of GQDs on the electronic characteristics of PTFE/MOs when electronic parameters change.

## 2. Materials and Methods

PTFE 100% hermetic seal (20 mm × 19 mm × 0.25 mm density 0.3 g/cm^3^) was from RICH, Italy. Copper sulphate pentahydrate (CuSO_4_·5H_2_O) was utilized to produce CuO nanoparticles. Glacial acetic acid, dimethyl sulfoxide (DMSO) (LabScan Ltd., Unit, Blackrock, Ireland) and sodium hydroxide (Fisher chemical, 97 percent, Suwanee, GA, USA) were utilized as solvents. This experiment made use of deionized (DI) Milli-Q water. All materials were utilized without additional purification.

### 2.1. CuO Nanoparticles Preparation Method

CuO nanoparticles were synthesized according to the usual precipitation procedure—1 M sulphate pentahydrate (CuSO_4_·5H_2_O) in 100 mL glacial acetic acid with stirring for 2 h at 70 °C. After the solution was completely dissolved, 2 M of Sodium hydroxide was added dropwise in 100 mL of DI water while stirring. The black precipitate was filtrated as well as washed using DI water, then dried at 80 °C for 24 h and later calcined for 2 h at 500 °C.

### 2.2. Preparation of the PTFE/CuO/G Nanocomposite

To start the preparation process, Graphene was prepared in a laboratory following the Hammer method, as indicated earlier in the literature under code (IFP-KKU-2020/10) [33]. PTFE was cut into microfibers to be used in the nanocomposite fabrication. 0.01 gm of CuO combined with 0.02 g of G were dissolved using (100 mL) DMSO solution, and then PTFE microfibers (70%) were mixed in the solution for 24 h with continuous stirring. The composite fibers were then removed and dried in air.

### 2.3. Characterization Techniques

For the intermolecular investigation of pure and composite materials, an Attenuated Total Reflection Fourier Transform Infrared (ATR-FTIR) spectrometer (Vertex 70, Bruker, Billerica, MA, USA) with a spectral range of 4000–400 cm^−1^ and a spectral resolution of 4 cm^−1^ was utilized. Furthermore, X-ray Diffraction (XRD) was used to determine the crystal structure and phase composition of samples using a Malvern Panalytical Empyrean 3 diffractometer (Malvern, UK). Furthermore, Field-emission Scanning Electron Microscopy (FESEM, Quattro S, Thermo Scientific, Waltham, MA, USA) was utilized to analyze the morphology of the produced samples.

### 2.4. Calculation Details

The GAUSSIAN 09 software (Gaussian, Inc.: Wallingford, CT, USA) was used to design model designs for four PTFE units and their interactions with MOs such as MgO, Al_2_O_3_, SiO_2_, TiO_2_, Fe_3_O_4_, NiO, CuO, ZnO, and ZrO_2_ at the Molecular Spectroscopy and Modeling Unit, National Research Centre, Egypt [34]. The HOMO/LUMO band gap energy, TDM, and MESP as contour were calculated for model structures using DFT theory at the B3LYP level with the LANL2DZ basis set [35,36,37]. 

## 3. Results

### 3.1. Characterization Result of the PTFE/CuO/G Composite

#### 3.1.1. FTIR Result

**Figure 1:**
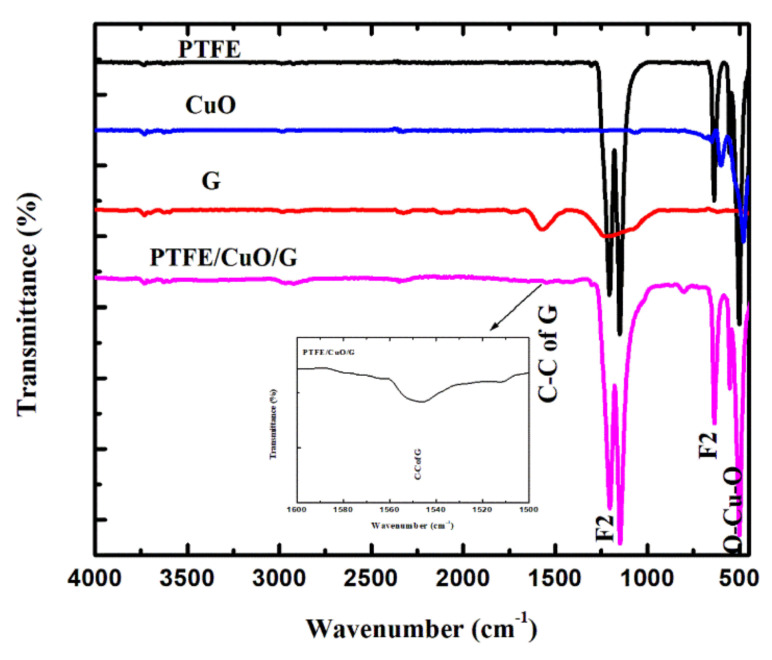
PTFE, CuO, G and PTFE/CuO/G transmittance ATR–FTIR spectra.

FTIR spectra for pure PTFE, CuO, G and PTFE/CuO/G composite are illustrated in Figure 1. The characteristic bands for pure PTFE microfibers are shown as only transmittance bands of F_2_ stretching at 1204 cm^−1^, 1152 cm^−1^, and 635 cm^−1^, respectively [38]. Moreover, CuO nanoparticles spectra displays a prominent band at 598 cm^−1^, indicating CuO generation [39]. The PTFE/CuO/G spectrum reveals the recognized bands of PTFE microfibers and even a new band at 1556 cm^−1^, reflecting the C−C of G with lower intensity in respect to a lower ratio and good distribution of the composite, and a CuO band which moved to a lower wavenumber at 553 cm^−1^, confirming the composite creation of PTFE, CuO, and G.

#### 3.1.2. XRD Result

**Figure 2:**
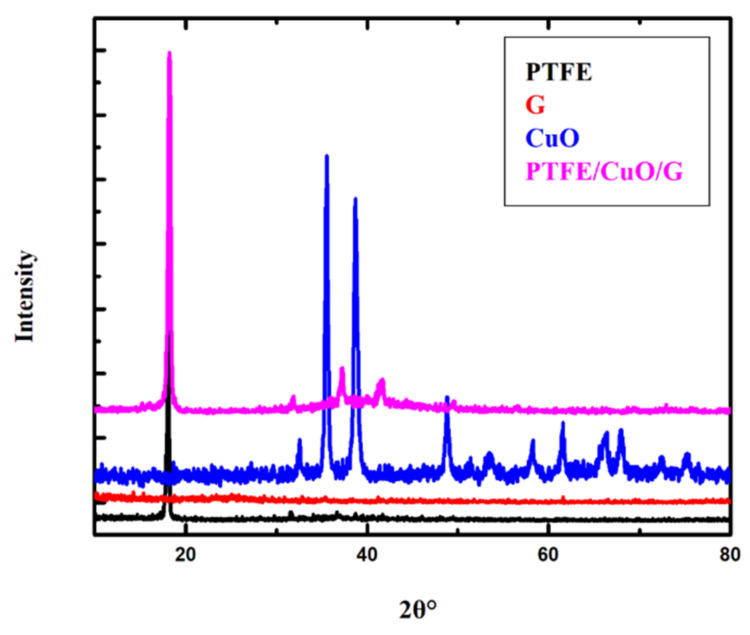
XRD diffraction pattern for PTFE, CuO, G, and PTFE/CuO/G nanocomposite.

As shown in Figure 2, the XRD pattern of PTFE microfibers was observed at around 2θ = 18.02° and 31.60°, relative to (100) and (110) diffraction plan [40]. In addition, the XRD pattern of monoclinic crystal CuO nanoparticles appearing around 2θ = 32.50°, 35.50°, 38.71°, 48.79°, 53.49°, 58.19°, 61.52°, 66.08°, 67.94°, 72.36°, and 75.11° which can be attributed to the reflection planes of (110), (−111), (111), (−202), (020), (202), (−113), (−311), (113), (311), and (−222), respectively [41]. Then, a characteristic diffraction peak of G was at about 2θ = 25.12°, which is related to the (002) reflection plan [42]. Finally, PTFE/CuO/G composite diffraction peaks were seen at 2 = 18.16°, 24.93°, 31.79°, 37.15° 41.49°, and 72.89° in relation to the (100), (222), (110), (107), (108), and (311) reflection planes. The intensity of the G peak was so small according to the interaction of CuO on the two G sheet surfaces, indicating that the PTFE/CuO/G nanocomposite was formed with high purity. Particle size calculation from X-ray diffraction and by considering the peak at degrees, average particle size was estimated by using the Debye–Scherrer formula [xx]: D = 0.9λ/β cos θ (1), where λ is the wavelength of the X-ray (0.15406 nm), β is FWHM (full width at half maximum), θ is the diffraction angle, and ‘D’ is the particle size diameter; thus, D = 111 nm as particle size. This would be more accurate in individual CuO because the composite would be on a micro scale, invading the limits of equations and accuracy, as estimated from the FESEM [43,44].

#### 3.1.3. SEM Result

**Figure 3:**
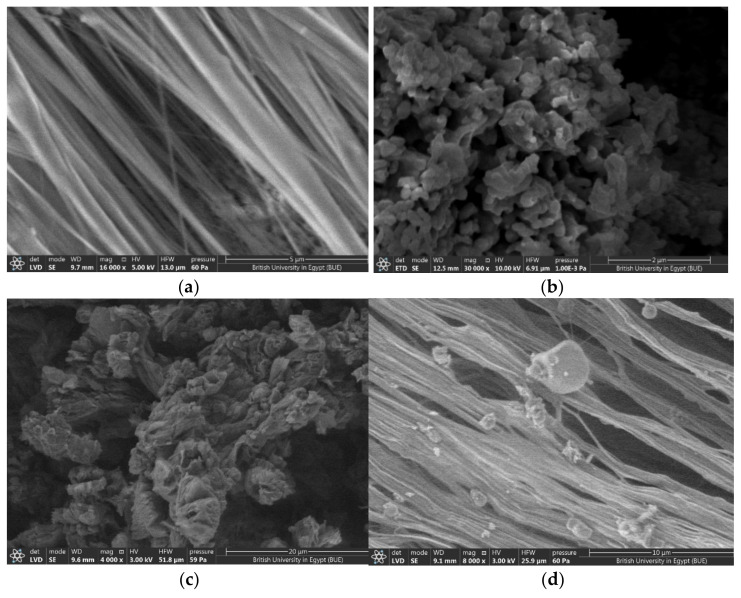
FESEM of (**a**) PTFE, (**b**) CuO, (**c**) G and (**d**) PTFE/CuO/G composite samples.

**Figure 4:**
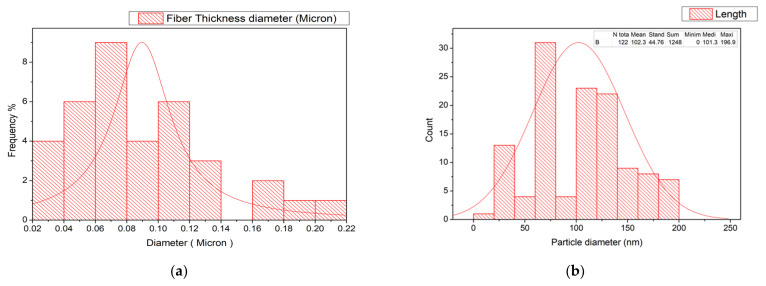
Histogram of the (**a**) fiber thickness and (**b**) CuO-NPs size distribution and curve of mean value.

Figure 3 shows an electron microscope image of the surface of PTFE microfibers, CuO, G, and PTFE/CuO/G composite. For a pure PTFE image, the diameters of the PTFE fibers were measured by using an image-analysis system consisting of an FESEM, a high-resolution monitor, and image-analysis by image j^®^ Program. The estimated diameter size is 100 nm. The thickness size distribution curve is indicated in Figure 4. The distribution curve indicates a narrow unimodal size distribution in the range from 20 to 220 nm with an average size of 100 nm [45]. For CuO-Nps, similar to the above analysis, the average particle size distribution was found to be in the range of (101–196 nm) with mean of 102 nm, as indicated in Figure 4b, ensuring the estimated value obtained by FESEM.

Small spherical CuO nanoparticles with homogenous distribution were illustrated in the morphological SEM image [46]. Moreover, the G SEM image illustrates agglomeration layers. As a result, PTFE/CuO/G illustrates uniform distribution of the quantities of CuO and G on the PTFE microfibers’ surface, which confirms the formation of the PTFE/CuO/G composite.

According to the experimental results of enhancing PTFE microfibers with CuO and G, a theoretical analysis of PTFE with different Mos, including MgO, Al_2_O_3_, SiO_2_, TiO_2_, Fe_3_O_4_, NiO, CuO, ZnO, and ZrO_2_, might be performed to study the change in electronic characteristics.

### 3.2. Designed Model Structures 

**Figure 5:**
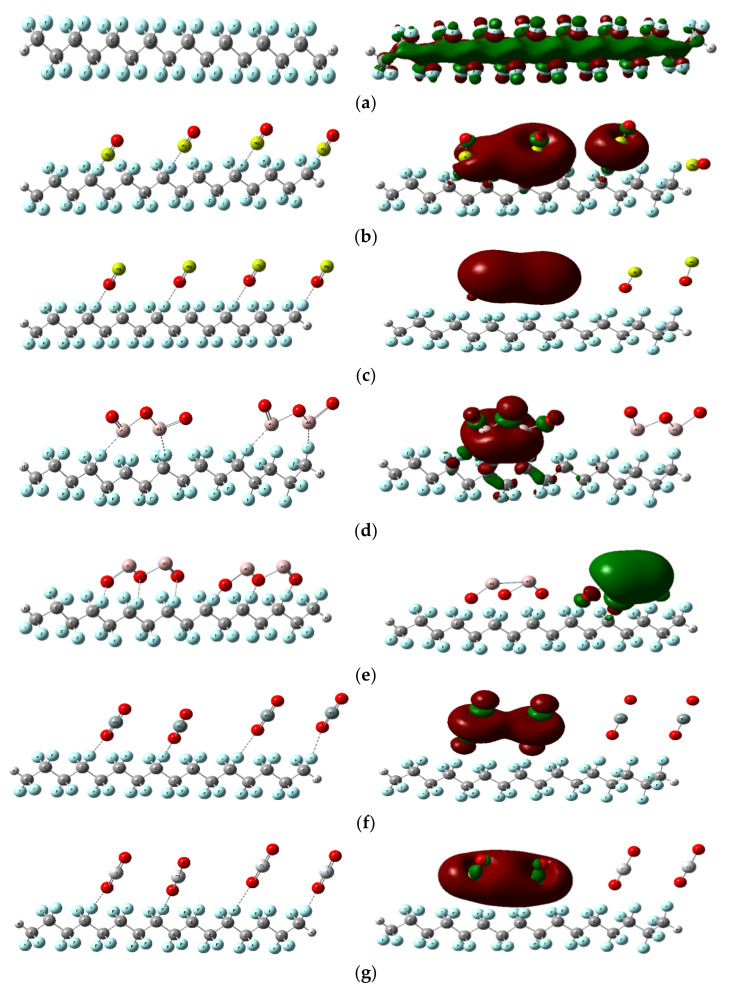

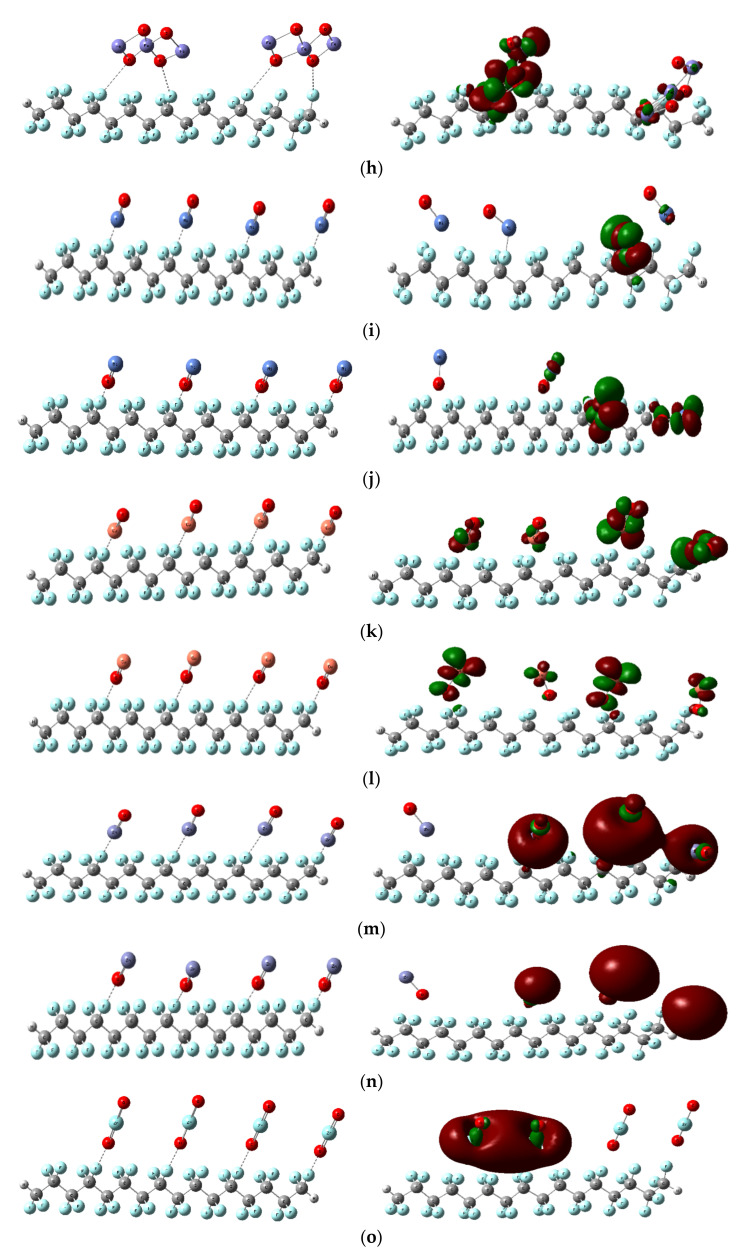
Optimized structure and HOMO/LUMO orbital distribution of PTFE interacting with different MOs using DFT:B3LYP/LANL2DZ as (**a**) PTFE, (**b**) PTFE/MgO, (**c**) PTFE/Omg, (**d**) PTFE/Al_2_O_3_, (**e**) PTFE/O_3_Al_2_, (**f**) PTFE/SiO_2_, (**g**) PTFE/TiO_2_, (**h**) PTFE/Fe_3_O_4_, (**i**) PTFE/NiO, (**j**) PTFE/ONi, (**k**) PTFE/CuO, (**l**) PTFE/OCu, (**m**) PTFE/ZnO, (**n**) PTFE/OZn, (**o**) PTFE/ZrO_2_.

**Figure 6:**
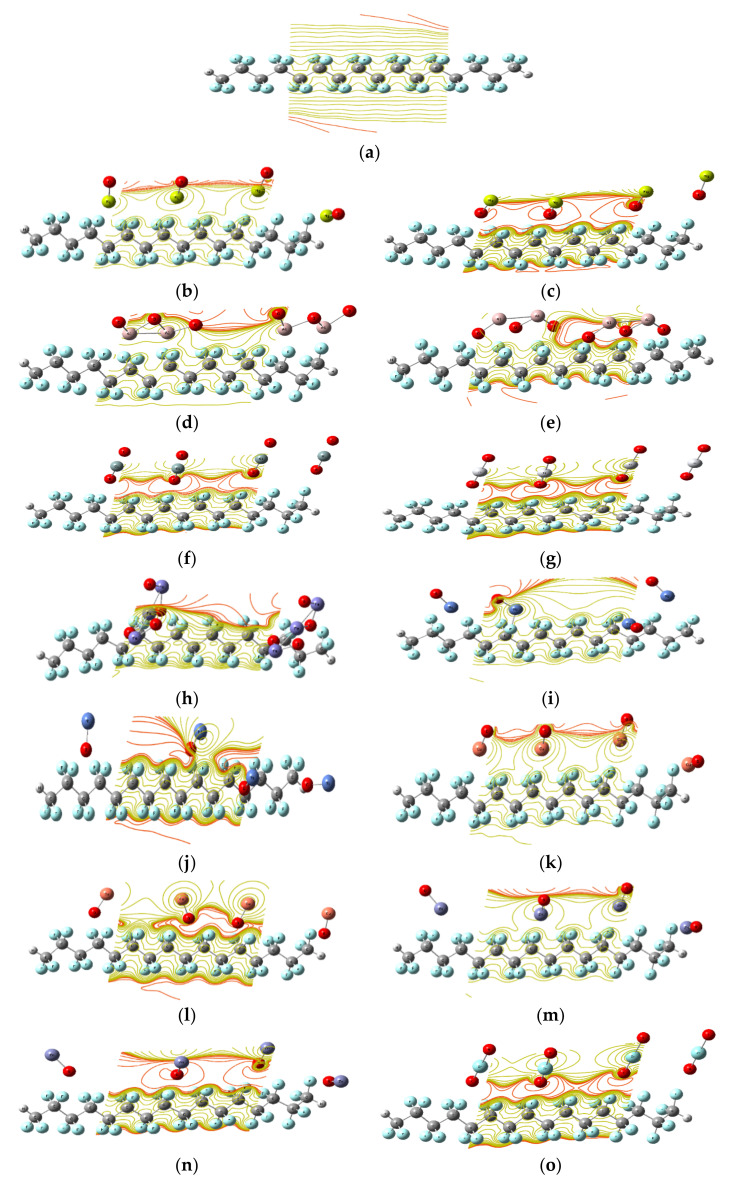
MESP mapping of PTFE interacting with different MOs using DFT:B3LYP/LANL2DZ as (**a**) PTFE, (**b**) PTFE/MgO, (**c**) PTFE/Omg, (**d**) PTFE/Al_2_O_3_, (**e**) PTFE/O_3_Al_2_, (**f**) PTFE/SiO_2_, (**g**) PTFE/TiO_2_, (**h**) PTFE/Fe_3_O_4_, (**i**) PTFE/NiO, (**j**) PTFE/ONi, (**k**)PTFE/CuO, (**l**) PTFE/OCu, (**m**) PTFE/ZnO, (**n**) PTFE/OZn, (**o**) PTFE/ZrO_2_.

**Figure 7:**
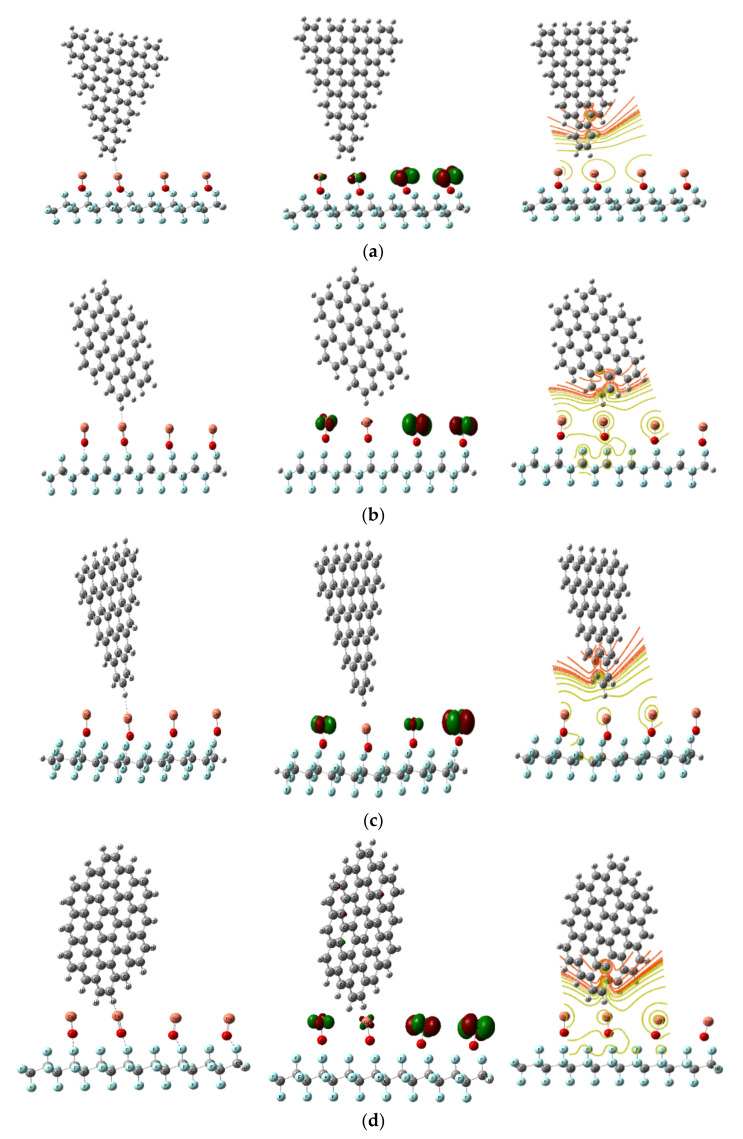
Model structure and calculated HOMO/LUMO orbital distribution and MESP mapping of PTFE/OCu reinforced with GQDs DFT:B3LYP/LANL2DZ as (**a**) PTFE/OCu/GQD ATRI C60, (**b**) PTFE/OCu/GQD AHEX C42, (**c**) PTFE/OCu/GQD ZTRI C46, (**d**) PTFE/OCu/GQD ZHEX C54.

Nanocomposites are of great interest currently for a wide range of applications. The addition of nanofillers to polymer matrices improves their main properties, leading to better electrical and optical mechanical functionality [47]. As a result, the model structure assumes four monomers of PTFE interacting with a variety of MOs, including MgO, Al_2_O_3_, SiO_2_, TiO_2_, Fe_3_O_4_, NiO, CuO, ZnO, and ZrO_2_. It is worth mentioning that for each metal oxide MO, there are two modes of interaction between the MO and the PTFE—one from the side of the metal atom and the other from the oxygen atom. Figure 5, Figure 6 and Figure 7 presents the PTFE; it first interacts with MgO and then Omg; this is repeated for all the studied MOs.

To follow up the reactivity of the PTFE’s interaction with MOs and graphene, the change in the HOMO/LUMO orbitals is mapped, followed by planning the MESP for those model structures. The values of the calculated HOMO/LUMO band gap energies and the total dipole moment are also summarized in Table 1 and Table 2.

#### 3.2.1. Interaction of PTFE with Different Metal Oxides

##### Distribution of HOMO/LUMO Orbitals

Four PTFE monomers were assumed to interact through the F atom, indicating that the active side of PTFE interacts through the F atom with numerous Mos, including MgO, Al_2_O_3_, SiO_2_, TiO_2_, Fe_3_O_4_, NiO, CuO, ZnO, and ZrO_2_. The distribution of HOMO/LUMO orbitals changed because of the interaction of PTFE with MOs, as seen in Figure 5. The HOMO/LUMO orbital dispersion is spread throughout the whole chain of the four PTFE monomers. When MOs interacted with the PTFE surface, HOMO/LUMO orbitals delocalized all over MO atoms. According to this, increasing TDM while lowering ΔE improved electrical characteristics, in addition to structure stability and reactivity [48]. Table 1 presents TDM along with band gap energy (ΔE) computed for all designed interactions. 

TDM were increased according to PTFE interactions with the proposed MOs (MgO, OMg, Al_2_O_3_, O_3_Al_2_, SiO_2_, TiO_2_, Fe_3_O_4_, NiO, ONi, CuO, OCu, ZnO, OZn, and ZrO_2_ from 00.000 Debye to 30.100, 27.449, 22.477, 12.709, 00.231, 04.722, 06.575, 19.260, 08.924, 20.532, 76.136, 22.524, 19.137, and 07.526 Debye, respectively. Simultaneously, band gap energy (ΔE) also decreased according to PTFE interactions with proposed MOs from 8.510 eV to 1.466, 1.327, 1.102, 0.407, 3.226, 1.366, 1.996, 0.579, 1.106, 0.506, 0.400, 1.909, 2.317, and 0.938, respectively. As a consequence, PTFE/OCu seemed to have the lowest band gap value, signifying an improvement in electrical, responsiveness, and durability properties.

##### Molecular Electrostatic Potential (MESP)

Figure 6 illustrates the MESP of PTFE and PTFE interactions with numerous MOs, such MgO, Al_2_O_3_, SiO_2_, TiO_2_, Fe_3_O_4_, NiO, CuO, ZnO, and ZrO_2_. MESP is another significant characteristic for understanding the electrical properties of chemical interactions. MESP is significant because it may connect between changes in total charge and the influence on physical and chemical characteristics for studied structures. The distribution of MESP on the molecule’s surface was illustrated by a map with the colors Red > Orange > Yellow > Green > Blue, with the red color representing the greatest charge zone, the blue color representing the lowest charge zone, and the green color representing zero charge zone [49]. The F atom was revealed to be the active side of the low reactivity of PTFE. The colour red intensified along the polymer chain’s up and down branches due to the F atom, which is an indication for the PTFE active side. Because of the influence of MOs, the red colour increased and relocated then around the oxygen atom of the MO, indicating an improvement in PTFE reactivity. According to the MESP observations, PTFE’s electrical properties increased, allowing it to be used in a wide range of applications.

##### GQDs Interaction with PTFE/OCu

According to earlier findings, the four GQD forms—ATRI, AHEX, ZTRI, and ZHEX—should be examined with PTFE/OCu. Owing to the GQDs’ features of large surface area and effective edge atoms, they have great interaction with the surrounding molecules [50]. The most electronically improved active structure is PTFE/OCu, so it was chosen to interact with the four GQDs forms, as illustrated in Figure 7. From Table 2, TDM of PTFE/OCu changed from 76.136 Debye to 20.421, 16.439, 39.124 and 17.571 Debye, while the band gap decreased from 0.400 eV to 0.480, 0.432, 0.206 and 0.433 eV for PTFE/OCu/GQD ATRI C60, PTFE/OCu/GQD AHEX C42, PTFE/OCu/GQD ZTRI C46, and PTFE/OCu/GQD ZHEX C54, respectively. From all data, PTFE/OCu/GQD ZTRI C46 is the most effective, stable, and novel in electrical properties and could be used in nanoelectronic devices.

## 4. Conclusions

This work combined both experimental and DFT:B3LYP/LANL2DZ calculations to gain better insight into the molecular structure of the studied polymer PTFE as well as its graphene and Mos-modified structure. 

Thus, the PTFE microfibers were reinforced with CuO and graphene G, then studied with some characterization techniques such as FTIR, XRD, and SEM. The FTIR results confirmed that the PTFE/CuO/G is formed as a composite structure, and the SEM image showed the uniform distributed of nanoparticles on the PTFE microfibers’ surface. 

DFT calculations as consulted to study PTFE interacted with various MOs. Throughout the calculations, the HOMO/LUMO orbitals were mapped. It could be concluded that MOs are responsible for reducing the HOMO/LUMO band gap by changing it from broad to small band gap semiconductor. 

The addition of various MOs to PTFE creates and controls a wide range of band gaps, allowing for several applications, including as a solar cell, sensor, and capacitor. 

Another important mapping throughout the calculations is the molecular electrostatic potential map, which indicated an active site starting from the PTFE step by step during the interaction with graphene as well as other MOs.

The structure is considered active as its calculated total dipole moment increased with a decrease in its HOMO/LUMO band gap energy. 

It could thus be concluded that CuO was the most effective MO to increase the electronic characteristics of PTFE. Thus, it was chosen to interact with the four GQDs forms, namely ATRI, AHEX, ZTRI, and ZHEX. 

The results confirmed that, the PTFE/OCu/GQD ZTRI C46 composite increases PTFE’s ability to perform in nanoelectronics devices, which are important in space applications.

## Figures and Tables

**Table 1:** Calculated TDM (Debye) combined with ΔE (eV) for PTFE interactions with supposed MOs (MgO, Al_2_O_3_, SiO_2_, TiO_2_, Fe_3_O_4_, NiO, CuO, ZnO, and ZrO_2_) using B3LYP/LANL2DZ.

Structure	TDM (Debye)	ΔE (eV)
PTFE	00.000	8.510
PTFE/MgO	30.100	1.466
PTFE/OMg	27.449	1.327
PTFE/Al_2_O_3_	22.477	1.102
PTFE/O_3_Al_2_	12.709	0.407
PTFE/OSiO	00.231	3.226
PTFE/OTiO	04.722	1.366
PTFE/Fe_3_O_4_	06.575	1.996
PTFE/NiO	19.260	0.579
PTFE/ONi	08.924	1.106
PTFE/CuO	20.532	0.506
PTFE/OCu	76.136	0.400
PTFE/ZnO	22.524	1.909
PTFE/OZn	19.137	2.317
PTFE/OZrO	07.526	0.938

**Table 2:** TDM (Debye) and ΔE (eV) of GQDs interaction with PTFE/OCu using B3LYP/LANL2DZ.

Structure	TDM (Debye)	ΔE (eV)
PTFE/OCu/GQD ATRI C60	20.421	0.480
PTFE/OCu/GQD AHEX C42	16.439	0.432
PTFE/OCu/GQD ZTRI C46	39.124	0.206
PTFE/OCu/GQD ZHEX C54	17.571	0.433

## Data Availability

All original measurements and data analysis of this work will be available when required.
